# Contributions of the Schistosomiasis Consortium for Operational Research and Evaluation (SCORE) to Schistosomiasis Control and Elimination: Key Findings and Messages for Future Goals, Thresholds, and Operational Research

**DOI:** 10.4269/ajtmh.19-0787

**Published:** 2020-05-12

**Authors:** Daniel G. Colley, Fiona M. Fleming, Sultani H. Matendechero, Stefanie Knopp, David Rollinson, Jürg Utzinger, Jennifer D. Castleman, Nupur Kittur, Charles H. King, Carl H. Campbell, Fatma M. Kabole, Safari Kinung’hi, Reda M. R. Ramzy, Sue Binder

**Affiliations:** 1 Schistosomiasis Consortium for Operational Research and Evaluation (SCORE), Center for Tropical and Emerging Global Diseases, University of Georgia, Athens, Georgia;; 2 Department of Microbiology, University of Georgia, Athens, Georgia;; 3 Department of Infectious Disease Epidemiology, Schistosomiasis Control Initiative, London, United Kingdom;; 4 Division of Communicable Disease Prevention and Control, Neglected Tropical Diseases Unit, Ministry of Health, Nairobi, Kenya;; 5 Swiss Tropical and Public Health Institute, Basel, Switzerland;; 6 University of Basel, Basel, Switzerland;; 7 Department of Life Sciences, Wolfson Wellcome Biomedical Laboratories, Natural History Museum, London, United Kingdom;; 8 Center for Global Health and Diseases, Case Western Reserve University, Cleveland, Ohio;; 9 Neglected Diseases Programme, Ministry of Health of Zanzibar, Zanzibar, United Republic of Tanzania;; 10 National Institute for Medical Research (NIMR), Mwanza Centre, Mwanza, United Republic of Tanzania;; 11 National Nutrition Institute, General Organization for Teaching Hospitals and Institutes, Cairo, Egypt

## Abstract

Herein, we summarize what we consider are major contributions resulting from the Schistosomiasis Consortium for Operational Research and Evaluation (SCORE) program, including its key findings and key messages from those findings. Briefly, SCORE’s key findings are as follows: i) biennial mass drug administration (MDA) with praziquantel can control schistosomiasis to moderate levels of prevalence; ii) MDA alone will not achieve elimination; iii) to attain and sustain control throughout endemic areas, persistent hotspots need to be identified following a minimal number of years of annual MDA and controlled through adaptive strategies; iv) annual MDA is more effective than biennial MDA in high-prevalence areas; v) the current World Health Organization thresholds for decision-making based on the prevalence of heavy infections should be redefined; and vi) point-of-care circulating cathodic antigen urine assays are useful for *Schistosoma mansoni* mapping in low-to-moderate prevalence areas. The data and specimens collected and curated through SCORE efforts will continue to be critical resource for future research. Besides providing useful information for program managers and revision of guidelines for schistosomiasis control and elimination, SCORE research and outcomes have identified additional questions that need to be answered as the schistosomiasis community continues to implement effective, evidence-based programs. An overarching contribution of SCORE has been increased cohesiveness within the schistosomiasis field-oriented community, thereby fostering new and productive collaborations. Based on SCORE’s findings and experiences, we propose new approaches, thresholds, targets, and goals for control and elimination of schistosomiasis, and recommend research and evaluation activities to achieve these targets and goals.

## INTRODUCTION

The Schistosomiasis Consortium for Operational Research and Evaluation (SCORE; see: https://score.uga.edu/) was funded by a major grant from the Bill & Melinda Gates Foundation (BMGF) to help answer the question: “What evidence will help neglected tropical disease (NTD) program managers to do their jobs better?” and then to provide some of this evidence.^1–3^ SCORE’s three objectives were focused on i) how best to do mass drug administration (MDA) with praziquantel (PZQ) and complementary interventions for gaining and sustaining control and elimination of schistosomiasis; ii) the development and evaluation of mapping, monitoring, surveillance, and diagnostic tools; and iii) synthesis and dissemination of the key SCORE findings to the World Health Organization (WHO) and governments of schistosomiasis-endemic countries through the Ministry of Health (MoH) NTD program managers. Encompassed within these three main objectives were a myriad of other projects and subprojects. During SCORE’s 10+ years of life, from late 2008 through mid-2020, these ranged widely from research on the elucidation of the *Schistosoma haematobium* genome—research that produced new insights that tried to evaluate drug resistance and led to better appreciation of hybrid schistosomes^4^—to the design and implementation of a community-developed teachers’ behavioral tool kit and curriculum for use in endemic areas.^5^ Over time, as the schistosomiasis community’s research and programmatic priorities changed and new questions emerged, SCORE simultaneously evolved as well, while retaining its focus on garnering evidence for uptake by NTD program managers.

As discussed in an earlier article in this supplement,^3^ when SCORE was conceived in 2008, results from the Schistosomiasis Control Initiative (SCI; see www.schisto.org), originally funded by the BMGF, demonstrated that the stated goal of morbidity control of schistosomiasis using MDA with PZQ could be achieved at a national scale by MoH programs.^6^ At that time, PZQ had to be purchased, thus limiting availability to many countries that lacked donor funding or a strong political commitment to domestic financing for preventive chemotherapy with PZQ. Although NTD programs had been established in many sub-Saharan African countries, the rolling out of the schistosomiasis control components of these programs was, as yet, limited.^7,8^ The strategies of those programs for schistosomiasis were based on the WHO guidelines, which focused on reducing severe morbidity—not the subtle morbidities that were much more prevalent in endemic countries^9^—and largely copied mass campaigns to control and eliminate lymphatic filariasis through preventive chemotherapy administered through MDA annually for at least 5–6 years.^10^ However, optimal strategies for schistosomiasis preventive chemotherapy, including delivery platforms or timings, were not clear, and there was little evidence for when or how a program should best assess the impact of MDA to inform whether a change in strategy was indicated.

Shortly after significant scale-ups in controlling morbidity due to schistosomiasis, interest was developing in the elimination of schistosomiasis.^11,12^ Donors and non-governmental organizations reasonably questioned whether it is feasible to eliminate schistosomiasis and, if so, when they could anticipate an endgame through their newly increased contributions and efforts.^13^

One stumbling block in determining when the move from sustained control to the interruption of transmission would be appropriate was the insensitivity of the available mapping and diagnostic tools for determining the prevalence and intensity of schistosome infections. The widely used and accepted microscopy-based Kato–Katz assay for *Schistosoma mansoni* and urine filtration assay for *S. haematobium* were sufficiently sensitive and highly specific when used in areas of high prevalence and intensity, especially at the onset of control programs.^14,15^ However, empirical evidence demonstrated them to be insensitive when infections were of light or moderate intensity.^16^ Therefore, other major SCORE objectives were the development and assessment of tools that would be adequately sensitive and specific for mapping, monitoring, surveillance, and assessing the impact of MDA for *S. mansoni* in areas of low prevalence and developmental studies on a highly sensitive and specific assay suitable for diagnosis of infections by all human schistosome species.

Summaries and lessons learned from SCORE’s research can be found in other articles in this supplement to the *American Journal of Tropical Medicine and Hygiene.* These summary articles contain references to the primary publications, which include more in-depth information and data presentations that we hope will be useful in informing future schistosomiasis control and elimination efforts, as well as assist in the formulation of continued operational research for schistosomiasis and other NTDs.

This article outlines the major findings and key messages from SCORE’s large field studies on gaining and sustaining control and elimination, as well as on the detection of infection. It will describe how these findings are informing new practical targets for use by programs and the development of new approaches to achieve these goals. It will also highlight SCORE’s role in establishing a cohesive schistosomiasis control community and how this is contributing to the recognition and addressing of interconnected future operational research needs that will be essential for the continued progress in the achievement of programmatic goals.

## SCORE’S KEY FINDINGS AND KEY MESSAGES FOR NTD PROGRAM MANAGERS

As summarized in the preceding articles in this supplement and the primary publications before them, SCORE’s “key findings” and “key messages” are summarized in Table 1. These key findings and key messages stemming from SCORE contain both good and bad news. The first good news is that regardless of the MDA strategy used in terms of frequency or platforms, if reasonable coverage is achieved (usually at least 75% coverage would be considered reasonable), the result will be an overall lowering of prevalence and a greater lowering of intensity of infection in approximately 70% of villages.^17^ The first bad news is that at least a third of the villages will fail to appreciably lower the prevalence and/or intensity, and thus, their schistosomiasis will be left uncontrolled. The reasons some villages fail to respond are, as yet, unclear. In SCORE data, MDA coverage in villages that did not respond, which we call persistent hotspots (PHSs), appeared to be as good as the MDA coverage in responder villages.^18,19^ We hypothesize that the force of transmission in PHS villages is such that soon after MDA, enough villagers are reinfected and propagate the schistosome life cycle almost to the pre-MDA baseline level of infection. This rapid recurrence of transmission is in contrast to lymphatic filariasis, where MDA lowers reinfection levels because of long-term elimination of microfilariae. In schistosomiasis, although the source of eggs is diminished by MDA, the source of the infectious form, cercariae coming from infected intermediate host snails, is not. Furthermore, because cure rates with PZQ are estimated to be 70–90%,^20,21^ even with excellent coverage, MDA will leave a proportion of adult worms and all juvenile worms alive. Rebound of prevalence of schistosomiasis is a well-documented challenge, even when recommended “elimination as a public health problem (EPHP)” levels of prevalence of infection intensity are achieved.^11^

**Table 1:** Summary of SCORE’s six key findings and related key messages

	Key findings of SCORE	Key messages of SCORE
1	Four years of school-based treatment and/or community-wide treatment MDA with PZQ—biannually, annually, or biennially—is effective in reducing average *Schistosoma* prevalence and the intensity of infection.	Biennial MDA through school and/or communities is sufficient to reach a programmatic goal of moderate prevalence of infection.
		Biennial MDA is insufficient to reach low prevalence of infection.
2	No SCORE MDA regimens eliminated *Schistosoma* transmission within 5–6 years.	MDA alone will not achieve a programmatic goal of elimination of transmission in most settings.
3	All MDA regimens in the SCORE gaining control and sustaining control studies left at least 30% of the study villages in all study arms as persistent hotspots. (PHSs) these PHS villages, by definition, failed to decrease as expected in prevalence and intensity following multiple years of MDA.	A programmatic goal to gain and sustain control of schistosomiasis in all villages receiving MDA should identify persistent hotspots following 2 years of annual MDA through an epidemiologic assessment.
	Monitoring outcomes to assess if villages are likely to be PHS versus responder villages is feasible as soon as a year after 2 years of annual MDAs.	On identification of PHSs, interventions should be adjusted, for example, more intensive MDA and/or complementary interventions to drive down the prevalence and intensity of the PHS. Continued research is needed to evaluate these options. Efforts can be maintained, adapted, or possibly decreased in responder villages.
4	If a village starts with ≥ 25% prevalence, then over 4 years, annual MDA is (i) more effective at reducing prevalence and intensity than 2 years of MDA (biennial) and (ii) leaves fewer PHSs than biennial MDA.	A program will be most effective at reducing prevalence and intensity of infection and lowering the number of PHS villages with annual MDA.
5	Both control of morbidity and elimination as a public health problem goal^28^ based on the percent prevalence of heavy infection are achieved through MDA.	Current WHO definitions of control of morbidity and elimination as a public health problem based on intensities of infection are inappropriate for determining success related to changes in infection.
	Often, in areas with established moderate-to-high prevalence, these goals defined by the prevalence of heavy intensity are fulfilled even before any MDA, and, thus, need to be redefined.	For programs to determine success of their efforts to control and/or eliminate schistosomiasis, new targets, which are evidence-based, urgently need to be defined.
6	Use of the point-of-care circulating cathodic antigen (POC-CCA) urine assay for *S. mansoni* finds more low-intensity infections in low-to-moderate prevalence areas than the standard parasitologic methods.	The POC-CCA urine assay for *S. mansoni* is an appropriate tool for epidemiologic assessments (i.e., mapping and impact assessment) in areas of low-to-moderate prevalence (5–40% by Kato–Katz) to prevent undertreatment.
	The POC-CCA yields some false positives, especially in areas where prevalence is extremely low (i.e., < 5%).	POC-CCA cannot be used as a diagnostic tool to determine interruption of transmission (elimination).

MDA = mass drug administration; PHS = persistent hotspot; POC-CCAs = point-of-care circulating cathodic antigen assays; PZQ = praziquantel; SCORE = Schistosomiasis Consortium for Operational Research and Evaluation.

Another area of good and bad news revolves around the findings by SCORE investigators and others that neither the Kato–Katz assay nor the urine filtration assay are able to detect a large proportion of people who have low-to-moderate intensity infections with *S. mansoni* or *S. haematobium*, respectively.^22,23^ It is clearly good news that assays such as the point-of-care circulating cathodic antigen (POC-CCA) urine cassette test are better able to identify areas with mostly low-to-moderate intensity infections. This becomes more important as the number of low-intensity-level areas increases as a result of multiple years of MDA or other interventions. Another piece of good news is that infection with very low levels of either *S. mansoni* or *S. haematobium* can be confirmed with high sensitivity using the up-converting phosphor-lateral flow circulating anodic antigen (UCP-LF CAA) assay.^24,25^ It is, however, unwelcome news that so many more people harbor schistosomes than previously assumed and, therefore, are at risk in terms of morbidity and continue to pose a threat to attempts at elimination because of the continued production of viable eggs, potentially continuing transmission.

## SCORE CONTRIBUTIONS TO NEW WHO GUIDELINES

The SCORE data that generated the findings noted earlier are now contributing to the development of new or revised WHO guidelines for schistosomiasis. These new guidelines, plus the political commitments and domestic and donor funding required to implement them, will help NTD program managers optimize their approaches to morbidity control and elimination. We anticipate that the SCORE findings will be particularly useful because they represent large studies across different schistosome species and epidemiologic settings.

The SCORE findings that we consider to be currently most pertinent to new guideline development are as follows: i) the use of the POC-CCA urine assay for detecting *S. mansoni* infection and its relationship with the Kato–Katz technique based on stool samples; ii) that PHSs do not respond to current recommendations for frequency and delivery platforms for MDA, are more common than previously thought, and can often be detected after two annual MDAs; and iii) the inappropriateness of current heavy infection intensity thresholds as cutoffs to define control of morbidity or EPHP. These SCORE key findings highlight and reinforce the work of WHO, as well as geospatial and mathematical modelers, epidemiologists, and others in emphasizing the need to conduct more timely and appropriate epidemiologic impact assessments and ongoing surveillance specific to schistosomiasis programmatic needs.

### Use of the POC-CCA urine assay for detecting *S. mansoni* infection and its relationship with the Kato–Katz technique based on stool samples.

It is clear that either the Kato–Katz or POC-CCA assay can be used in high-prevalence areas but that the Kato–Katz assay is not adequate to measure prevalence in low-to-moderate *S. mansoni* intensity areas.^16^ This has been demonstrated in multiple epidemiologic studies, including several by SCORE, and in a major modeling exercise commissioned by the WHO, which included all available data to compare the Kato–Katz assay and the POC-CCA assay in endemic areas ranging from very low to high prevalence. Modeling of these multiple studies has established a usable nonlinear relationship between the prevalence found by these two assays.^26^ Thus, as prevalence thresholds for defining program actions are revised, there are now generally agreed-upon cutoffs for both of these assays for *S. mansoni*, providing flexibility for disease control programs.

In the SCORE Zanzibar elimination study^27^ embedded in the Zanzibar Elimination of Schistosomiasis Transmission (ZEST) program a very low prevalence setting where most of those infected harbored very light intensities of *S. haematobium* infections, it was also seen that the urine filtration assay to detect eggs and hematuria reagent strips to detect microhematuria had a low sensitivity to detect infections with less than five eggs per 10 mL of urine.^23,25^ Currently, efforts are underway to develop and evaluate a new POC-CAA test. Should it be successful and appropriately validated, it should find its way into the WHO guidelines in an expedited manner.

### Persistent hotspots do not respond to current recommendations for frequency and delivery platforms for MDA, and are more common than previously thought.

SCORE data have clearly demonstrated the pervasiveness of PHSs. Current guidelines suggest that decisions about MDA be guided by an initial prevalence assessment, often at a limited number of sentinel sites, with reevaluation of prevalence after 5 years. SCORE data indicate that PHSs can often be detected after two annual MDAs. Reassessment of the impact of MDA before the current 5-year recommendation could ensure that PHSs receive the more intensive interventions they need and that places that have responded well to MDA have their treatment regimens titrated appropriately.

### The inappropriateness of current heavy infection intensity thresholds as cutoffs to define control of morbidity or EPHP.

**Figure 1.**
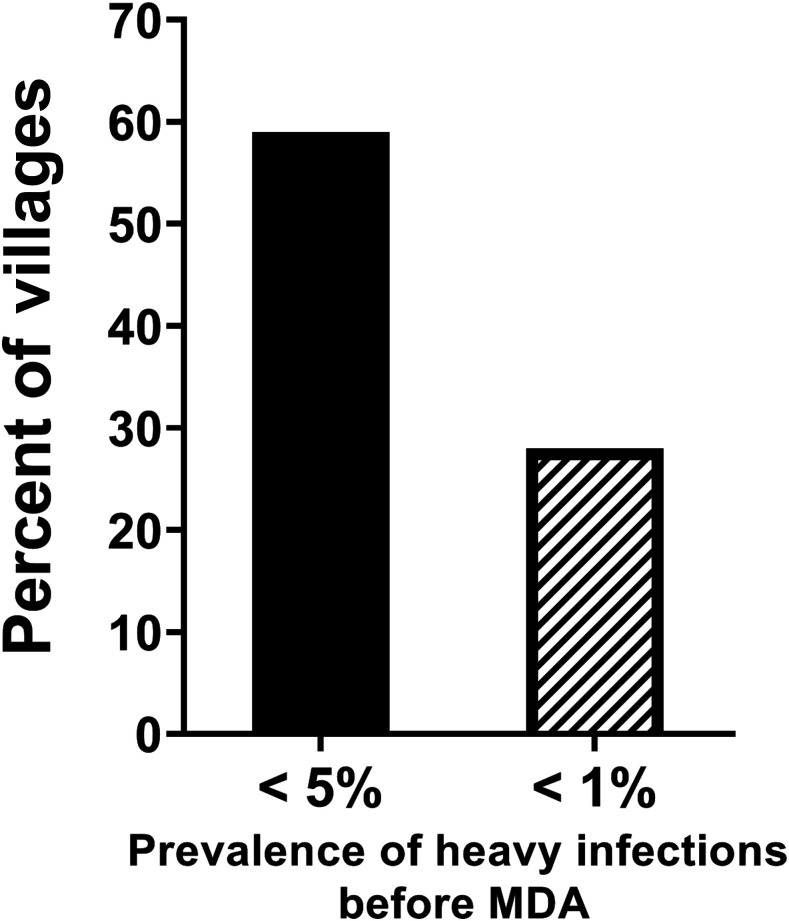
Percentage of villages that would have been classified as controlling morbidity (< 5% heavy infections [≥ 400 *Schistosoma mansoni* eggs per gram of feces] or having eliminated schistosomiasis as a public health problem (< 1% heavy infections) before any mass drug administration (MDA), in Schistosomiasis Consortium for Operational Research and Evaluation studies in 294 villages in high prevalence areas, in Kenya and Tanzania.

The WHO goals of achieving morbidity control and EPHP were based on decreasing heavy infections (≥ 400 eggs per gram of feces for *S. mansoni* and ≥ 50 eggs per 10 mL of urine for *S. haematobium*) to the prevalence levels of < 5% and < 1%, respectively.^28^ These thresholds were based on a limited number of early studies on severe schistosomiasis-related morbidity and not the kinds of nonspecific, more subtle, functional morbidities demonstrated to occur at levels of infection common in endemic areas today. Therefore, success in achieving these thresholds would leave many infected children—who are experiencing functional morbidity—untreated. Based on SCORE data from 294 villages in its gaining control study villages with a starting prevalence of ≥ 25% in *S. mansoni* areas in Kenya and Tanzania, 59% of villages had achieved the currently stated goal of control of morbidity before even starting MDA (Figure 1). Likewise, 28% of the villages had already achieved the currently stated goal of EPHP before their first MDA (Figure 1). Despite having already achieved these intensity-based thresholds, these villages had many children with infections. This emphasizes that the use of prevalence of heavy infections thresholds to determine whether adequate control of morbidity or EPHP has been achieved is inappropriate.

Instead of using intensity-based measures for cutoffs, we recommend using prevalence alone to determine targets for changes in strategies. This would eliminate the need to quantify egg output; rapid diagnostics, such as POC-CCA urine cassette assay^29^ and urinary dipstick for the detection of hematuria,^30^ would be sufficient for programmatic use and decision-making. If easily quantified proxies are developed that in fact reflect levels of morbidity, then a shift to use those measurements would be appropriate, as they would actually reflect whether morbidity was being controlled.

## CONTRIBUTIONS BY OTHER SCORE FINDINGS

In addition to the major impact findings and messages of the SCORE treatment and diagnostic studies, multiple other SCORE studies have provided new insights and data on a wide variety of schistosomiasis control- and elimination-related topics. These messages and findings are now contributing to the design of new research, guideline development, and program decision-making.

### Schistosome population genetics.

The schistosome population genetic studies were a component of both the gaining control studies and the Zanzibar elimination study. These studies examined the population genetic structure of schistosome populations following multiple community-wide MDAs (four MDAs over a 5-year period and 12 MDAs over a 6-year period in gaining control studies and the Zanzibar elimination study, respectively). As the basic SCORE design did not permit short-term pre- versus post-PZQ data collection (as any infected individual would have to be reoffered treatment), combined with the current lack of molecular markers specific to PZQ resistance, no direct assessment of potential drug resistance could be incorporated.^31^ Nevertheless, key findings of the population genetic analyses performed suggested that high levels of gene flow and mixing of the parasite populations between neighboring sites were likely to dilute any effects imposed by the SCORE treatment arms. Furthermore, significant inherent differences in parasite fecundity were observed, independent of current treatment arm, but potentially of profound impact in terms of maintaining high levels of ongoing transmission in apparent PHS sites. In addition, these investigations yielded new findings about the composition and distribution of naturally occurring *S. haematobium*/*Schistosoma bovis* hybrids across Niger.^32,33^ Such data and tools developed with SCORE support could prove useful in evaluating potential animal reservoirs that could perpetuate transmission.^31^

All new schistosome DNA sequences generated from this work have been deposited in the NCBI GenBank open-access sequence repository for access by other researchers. Similarly, through donations of snail and parasite materials, SCORE has contributed to the establishment and continuation of the Schistosomiasis Collection at the Natural History Museum (SCAN), which currently assures broad availability of schistosome genetic materials to any investigators for conducting additional genomic studies across *Schistosoma* species.^34^

### Tools for mapping and diagnosis at different levels of prevalence of infection.

In the area of diagnostics, SCORE has contributed substantially to the evaluation and acceptance of the POC-CCA assay for mapping *S. mansoni* as described earlier and as summarized in the article by Colley et al.^35^ in this supplement. In addition, SCORE has also contributed to a more complete understanding of the limitations of the POC-CCA assay in areas of very low to zero prevalence, such as some formerly endemic districts in the Nile Delta in Egypt and across St. Lucia in the eastern Caribbean Sea. In Egypt, children testing negative with Kato–Katz who were POC-CCA trace or 1+ positive had daily stools collected over a 30-day period. Based on Kato–Katz and miracidia hatching tests of all stools^36^ and subsequent repeated treatment studies,^37^ it was determined that they were not likely to be contributing to transmission nor suffering egg-induced morbidity.

SCORE also invested substantially into the development of the highly sensitive and genus-specific UCP-LF CAA assay.^38^ This tool should play an important role as a highly sensitive and specific diagnostic test and for verification of elimination of schistosomiasis. This assay has now been used many times, including in the SCORE studies within the ZEST program on Zanzibar, where it demonstrated much higher levels of *S. haematobium* than measured by the widely used urine filtration method.^25^ The UCP-LF CAA assay was also used in Burundi^24^ and Rwanda, where it contributed to our understanding of the strengths and limitations of the POC-CCA, and in numerous other studies.^38^

### SCORE’s role in refocusing attention on the importance of snails in transmission.

SCORE has contributed substantially to a rejuvenation of interest in the control of the vector snails and the potential of monitoring snails in determining the force of infection.^39^ Interest has also developed in xenomonitoring as a potential means for verification of elimination of transmission. The discipline of malacology and snail control, once the mainstay of schistosomiasis control,^40^ became essentially moribund on the advent of oral therapy with oxamniquine and then the major increased reliance on PZQ for widespread drug-based control. However, as implemented within the SCORE Zanzibar elimination study, which compared different interventions for schistosomiasis elimination, focal snail control in human–water contact sites, where snails were found, did not reveal any added benefit of mollusciciding over intervention by biannual MDA alone.^41,42^ However, because of a relatively small sample size, the fact that neighboring communities were randomized to different interventions (e.g., a community assigned to snail control could be near one receiving only MDA), and other design issues, these data are not conclusive regarding whether addition of snail interventions could indeed complement other interventions in elimination settings such as Zanzibar. A cluster-randomized trial supported by SCORE in the northern and central parts of Côte d’Ivoire is designed to provide insights into the potential role of snail control in the elimination of *S. haematobium* in areas of seasonal transmission,^43^ but the analyses of the data are not yet complete. Indeed, snail control may yet prove to be a needed component for some control and elimination programs, if applied to non-randomized, larger areas and potentially with more intensive coverage.

By holding SCORE snail control–focused meetings and through SCORE projects that included snail control,^39,42^ (Dr. Mamadou Ouattara, personal communication) snail population analyses, and xenomonitoring of schistosomes in snails,^39^ there has been a renewed cognizance of the potential utility of snail control in gaining control, perhaps in PHS, and sustaining control, as well as when moving toward elimination. In part because of this renewed interest, the WHO has held two malacology training sessions in sub-Saharan Africa in hopes of bringing back the much needed malacological expertise. In addition, the WHO has produced a new manual on snail control for use by present-day NTD programs.^44^

In addition, based on the interest exhibited in snail-predatory prawns that might provide an effective, natural snail control intervention,^45^ SCORE supported a descriptive study comparing the abundance of prawns and of schistosomiasis intermediate host snails and the local prevalence of schistosome infections in people living along two river systems in the southeastern part of Côte d’Ivoire. This study found an inverse relationship between prawn numbers and the number of intermediate host snails in given locations, but neither was correlated with the prevalence of schistosome infections in people.^46^ Another SCORE snail-predator study evaluated the residual impact of the introduction 20 years previously of a different snail predator, the Louisiana red swamp crayfish, into endemic areas in Kenya. Neither human schistosome-transmitting snails nor these crayfish were found in the areas of the earlier experimental releases. In some other areas in Kenya, there were thriving crayfish populations but only rare or no populations of schistosome intermediate host snails (E. S. Loker, personal communication). The relationships between aquaculture (of crayfish-eating catfish), agriculture (with high levels of snail- and crayfish-toxic pesticides), and snail habitat need to be considered in regard to schistosome transmission.

### SCORE’s role in contributing real-world data to the modeling of schistosomiasis control and elimination.

The SCORE large-scale trials have provided invaluable data for modelers studying the outcomes that can be expected following implementation of different interventions.^47^ Because the populations studied were very large, similar protocols were used in multiple SCORE sites, and data were collected not only on school-age children but also on adults and children entering school for the first time; the SCORE data have been used to develop and rigorously validate models, for the development of methods, and to make predictions. Through collaboration with the BMGF-funded NTD Modeling Consortium and through separately funded SCORE modeling efforts, a new consensus has emerged regarding the special characteristics of schistosomiasis control programs, such as PHS, and the changes needed in terms of program targets and implementation. Calibration of models against SCORE results confirmed the high risk of programs plateauing and potential rapid rebounds of prevalence when MDA is used alone in moderate- and high-prevalence communities. It was determined that the snail phase of transmission is not a linear function of community egg output, so that reductions in mean intensity of human infections will not reliably reduce the overall force of infection. The utility of having accurate coverage data and of knowing adult and preschool infection prevalence levels was reinforced. The current analysis focusses on the timing and frequency of impact assessment for optimal MDA intervention and on the added cost-effectiveness expected when environmental changes, including through snail control and/or water and sanitation programs, are added.^43,48^

### Rapid answers project (RAP).

It should also be remembered that research does not necessarily mean new protocols and projects. The data may already be there, but simply need further comprehensive analyses for use in decision-making. Whenever this can be done effectively, it will save both time and money. From the beginning, SCORE was charged with the concept of assembling and synthesizing findings that, together, might make a strong enough statement to impact policy and interventions without the need for further expensive field studies. This has been the concept behind SCORE’s RAPs.^49^ Through meta-analyses and systematic reviews, King and his group have developed seven RAPs that have covered a wide variety of aspects of schistosomiasis research and/or control.

### How has the SCORE program contributed to more cohesiveness in the schistosomiasis field-oriented community?

The first year of SCORE was spent developing and improving protocols to identify the most pertinent operational research questions that could provide findings relevant for NTD program managers and policy-makers. To do this, SCORE held seven meetings with participants with diverse areas of expertise across many aspects related to schistosomiasis control and elimination.^3^ These meetings established foundations of broad interactions among investigators and furthered mutual trust. Bringing together investigators and implementers, many of whom do not normally get together, fostered in-depth exchanges of opinions that contributed greatly to the development of the SCORE research program and the actual protocols pursued within SCORE. Eventually, SCORE made sub-awards to more than 60 investigators in 15 countries in Africa and another eight countries in Europe and the Americas. The research pursued through these sub-awards was primarily undertaken through partnerships between African and European/American institutions.

Each year from 2010 to 2018, SCORE held an annual meeting where most of these researchers, program managers, public health implementers, and some of their junior colleagues gathered at the University of Georgia in Athens to discuss progress and pitfalls of the SCORE studies and their relationship to the general field of schistosomiasis. These SCORE annual meetings yielded both in-depth critiques of the SCORE studies and broad discussions of control and elimination efforts related to schistosomiasis. The meetings always involved the SCORE Advisory Committee and WHO participation and often included others from the broader NTD community, including donors. Likewise, SCORE’s staff, investigators, and intervention implementers were consistently included by the WHO in their meetings that dealt with schistosomiasis. The 10+ year continuing dialog fostered among the SCORE “family,” with inclusion of others from the NTD community and beyond, has led to a series of new collaborations and a level of familiarity, camaraderie, and mutual trust that was, for the most part, previously missing. The emergence of this consortium of multiple investigators and implementers who could criticize, mourn, and laugh together was, we believe, highly productive and has acted as a prelude for the much needed Global Schistosomiasis Alliance (GSA; https://www.eliminateschisto.org) that is now leading the way in coordinating the schistosomiasis research, control, and elimination community in support of the WHO’s NTD department and regional WHO offices.^50^

## HOW ARE NTD PROGRAM MANAGERS ALREADY USING SCORE’s RESULTS?

Within sub-Saharan Africa, in general, NTD program managers follow WHO’s guidelines to develop their control and elimination programs, which includes schistosomiasis. However, as guidelines are generic, some level of innovation outside the WHO guidelines is at a national program manager’s discretion to ensure programs are tailored to the needs of their target populations. In addition to SCORE data contributing to new, evidence-based WHO guidelines for schistosomiasis (see the aforementioned text), some national programs have begun to use SCORE data on mapping and monitoring using the POC-CCA assay and identifying the risk of PHS within their broader national programs.

Because of the perception, and indeed reality, that POC-CCA is an easier and more sensitive mapping tool to use in field surveys for the diagnosis of *S. mansoni* than the collection of stool for the Kato–Katz assay, there has been increasing demand from countries to use it within program settings. National NTD programs in Angola, Burundi, Côte d’Ivoire, Ethiopia, Namibia, Rwanda, and Uganda, among others, have used POC-CCA for determining the prevalence of infection within implementation units and subsequent treatment strategies. Subsequently, several programs have tracked treatment strategy impact through sentinel sites and other sampling schemes using POC-CCA as the main monitoring tool.^51,52^

The Uganda NTD control program in 2012–2013 used POC-CCA to provide 112 subcounties with estimates of prevalence of infection to inform their treatment strategy. These subcounties were either treatment-naive, as historic data and/or local knowledge determined them to be less than the national 20% threshold for MDA, or had fallen to less than the 20% threshold for MDA following ≥ 6 rounds of MDA with PZQ.

The 20% threshold used by the Ugandan NTD control program was derived from the 1998 WHO guidelines based on Kato–Katz assays.^53^ By this time, several SCORE publications had demonstrated the increased sensitivity of POC-CCA, plus reduced time in sampling.^54–57^ Thus, by using the POC-CCA for subcounty mapping, the national program was able to increase the numbers of schools sampled within a subcounty, which optimized evidence on the distribution of infection and the precision around the prevalence estimate within that subdistrict implementation unit. In light of minimal guidance available on how to interpret the POC-CCA results into treatment frequency, the national program used existing evidence to guide them from both in-country associations between POC-CCA and Kato–Katz prevalence, from a subset of the subcounty data, and SCORE data.^29^

Subdistrict mapping, like that used within Uganda, or at even lower level administrative units (i.e., parish, village, and clusters of villages), frequently termed precision mapping,^58^ is increasingly being performed within countries. District-level baseline mapping is sufficient to reduce under- and overestimation of treatment needs at the beginning of a program.^59^ However, as programs mature, the risk of infection reduces, and because of the heterogeneous distribution of *Schistosoma* infection, finer scale mapping is required to optimize the use of scarce resources and determine PHSs. The use of POC-CCA will be a critical tool within these more precise mapping surveys.

Mapping for PHSs requires a more explorative approach within areas that fail to respond. This may differ from the existing standard protocols for identification of schistosomiasis-endemic areas. Implementation units, which are suspected to have PHS, should be subjected to precision mapping, which is sufficiently granular to enable determination of village-level differences in response to treatment. The village or a cluster of a few villages identified to harbor a PHS can then be subjected to a more rigorous, comprehensive package of interventions. Strategies for control and elimination of schistosomiasis in such areas, which fail to respond, will include increased coverage and frequency of MDA; better deployment of water, sanitation, and hygiene; and better mainstreaming of behavior change communication targeting all stakeholders, including policy-makers and leaders, program implementers, community members, and those involved in snail control.

## ADDITIONAL RESEARCH NEEDS BEYOND SCORE

Operational research should be guided and focused on prespecified goals and targets. The goals of control, EPHP, and interruption of *Schistosoma* transmission can be seen as a spectrum, starting in areas with high prevalence and proceeding to those where zero prevalence is achieved and maintained through continued monitoring and surveillance. The tools and approaches used must change along that spectrum, and the operational research needed to evaluate and establish those tools and approaches also needs to change as new findings become available. However, when one can anticipate a future need further down the spectrum, it is imprudent to only do research on immediate needs. Such an approach means you will not be ready for the next step when you get there. Instead, the time to address future needs by basic and more directly transferable, operational research is well before the fruits of that research are needed. Research takes time, and when done well almost always leads to more questions and the need for more research. This is, of course, almost always true of basic research, but it is also often true of focused operational research.

The SCORE studies answered many questions and raised new ones, for example, by showing the existence of PHSs in all of the large SCORE field studies, regardless of what MDA strategy was used. This then involved the joint research questions of how to identify PHSs as early as possible and what to do about them.^18^ We believe that perhaps the most pressing overall needs, based on SCORE findings and those of collaborating modelers, can be summarized as follows: (i) the need to develop more sensitive and specific mapping, monitoring, and surveillance POC tools for low prevalence and intensity settings; (ii) the need to develop useful mapping and monitoring strategies to address the focal nature of schistosome transmission and identify PHS; and (iii) the need to develop effective intervention and adaptive strategies for dealing with both PHS and low-prevalence settings.

Furthermore, it must be emphasized that strategies and targets should be based on the stated goals of a program, and these goals should change over time based on the progress of the program. Operational research should be guided by this spectrum of goals and targets established by the NTD program. Table 2 lists potential operational research and tool development needs suggested to address the different goals that a program might aspire to, depending on their progress across the spectrum of control, control of morbidity, EPHP, and interruption of transmission. The addition of a goal entitled “gaining control” has been included as the initial-level goal. Although it is not a stated WHO goal, this is done for programs that primarily aspire to stay on top of their schistosomiasis problem and avoid hyperendemic situations in their countries. Furthermore, because it is currently a major challenge to define morbidity control, gaining control to a given prevalence level may be an acceptable goal.

**Table 2:** Operational research, evaluation, and tool needs, based on programmatic goals

Programmatic goal	Programmatic research and evaluation needs	Other research and assay needs
Gaining control*	Improved approaches to mapping, taking focal nature of schistosomiasis into account	Sensitive, specific field assays for low-to-moderate levels of *S. haematobium* infection
	Develop methods for early identification of persistent hotspots (PHSs) and responder villages (either before mapping or after a limited number—often as few as two—annual MDAs) and adaptive strategies	
	Improve efficiency and effectiveness of mass drug administration by defining the following:	
	Age-groups that need treatment, which may vary by PHSs versus responder villages and other factors	
	How to achieve high coverage	
	How to reach persistently noncompliant and persistently unreached individuals	
Morbidity control†	All of the items under “gaining control”	*S. haematobium* assay, as described under “gaining control”
	Assess the utility of making schistosomiasis testing with dipsticks and/or point-of-care circulating cathodic antigen assay and praziquantel available in health centers	Develop proxies for measuring attributable fraction of morbidity due to schistosomiasis in a field-applicable manner
		Determine the potential morbidity from hybrid schistosomes
Elimination as a public health problem‡	Assess test, treat, track, test, and treat strategies (5T), for example, testing in schools to determine if prevalence is low and, if so, providing follow-up assessment of positive cases for family members or others who potentially share transmission sources instead of MDA (which would treat many uninfected)	Develop and evaluate highly sensitive and specific point-of-care assays for *S. haematobium* and *S. mansoni* when prevalence is 5% or less
	Assess contributions of interventions other than MDA, such as snail control, sanitation and clean water provision, and behavioral change interventions to achieve and/or maintain this level	Based either on (i) the proxies developed to actually measure morbidity or (ii) an arbitrary level of prevalence considered to eliminate schistosomiasis as a public health problem‡ and decide on a clear and measurable definition of elimination as a public health problem
	For snail control, this includes evaluating such questions as the optimal timing and frequency of mollusciding and ways of delivering molluscicides (e.g., spraying versus drip-feed versus slow release compounds) and the potential for community engagement in environmental interventions like clearing vegetation	Determine if hybrids pose a threat for continued transmission
	For behavior change, this includes using interventions developed using human-centered design and other community-engaged approaches	Determine the monitoring sampling schemes to be used for surveillance of humans and snails and the frequency that they will be used
Interruption of transmission§	Develop and evaluate a transmission assessment survey for schistosomiasis	Test surveillance-response approaches and their integration into local health systems
		Develop and evaluate diagnostic assays for surveillance and confirmation of human and snail infections once elimination is achieved and detectable prevalence of both is zero
	Implement protocols for surveillance response with appropriate diagnostic assays and response interventions to ensure elimination is maintained	Determine if animal reservoirs are a major contributor to transmission to humans

MDA = mass drug administration; POC-CCAs = point-of-care circulating cathodic antigen assays.

* Arbitrarily defined as achieving and maintaining prevalence levels at or less than 25% by Kato–Katz/urine filtration or 50% by POC-CCA.

† Arbitrarily defined as achieving and maintaining prevalence levels at or less than 15% by Kato–Katz/urine filtration or 30% by POC-CCA.

‡ Arbitrarily defined as achieving and maintaining prevalence levels at or less than 2% by Kato–Katz/urine filtration or 10% by POC-CCA.

§ Defined as zero transmission based on highly sensitive and specific survey diagnostics and/or zero anti-schistosome antibodies in 6- to 10-year-old children for three consecutive years, that is, passing a defined transmission assessment survey for 3 years.

## ENSURING SCORE DATA AND SPECIMENS ARE AVAILABLE FOR FUTURE COMMUNITY USE

From the beginning, SCORE was committed to ensuring that the knowledge gained be broadly disseminated to the scientific community and made accessible to the people and programs at most need within the schistosomiasis-endemic countries of the world. All SCORE investigators agreed to these goals and to the University of Georgia Research Foundation, Inc./BMGF Global Access Policy.

As SCORE was being conducted, processes were put in place whereby individuals and organizations could request access to SCORE data even before the data were published. Such requests were referred to the investigators, who were encouraged to be liberal in their sharing. Because SCORE research had to be published as open access, the underlying data for all SCORE publications are available.

As the SCORE secretariat completes its role, plans have been put in place to ensure continued access to the large amounts of data collected in the SCORE field studies. SCORE has put epidemiologic data, including demographic and parasitologic results, from the gaining control and sustaining control studies, morbidity cohort study, Zanzibar and seasonal elimination studies, Rwanda and Burundi surveys, and the five-country POC-CCA study into standardized formats, termed the SCORE Unified Datasets (SUDSs). Through its relationship with the Kissinger Research Group and EuPathDB at the Center for Tropical and Emerging Diseases at the University of Georgia and at BMGF, SCORE was introduced to ClinEpiDB (https://clinepidb.org/ce/app/), a relatively new, highly interactive repository for epidemiologic data. Working closely with the ClinEpiDB staff at the University of Pennsylvania, all the SUDS files will be loaded onto the ClinEpiDB platform by mid-2020.

Other non-epidemiologic SCORE data will not be housed on ClinEpiDB but will be standardized and made accessible on an appropriate site. For example, snails collected in SCORE studies are stored at SCAN in London, United Kingdom. The data from the snail studies will be made available as a darwincore archive to the Global Biodiversity Information Facility, and as indicated earlier, new schistosome DNA sequences generated from SCORE studies are deposited in the NCBI GenBank open-access sequence repository.

The SCORE program was conceived, developed, and implemented, and it then evolved to try to meet the evidence-based needs of NTD program managers in their efforts to control and eliminate *S. mansoni* and *S. haematobium* infections. To do this, it focused on comparative uses of the primary intervention tool at the time, preventive chemotherapy with PZQ, with forays into combined interventions involving snail control and behavioral change. Furthermore, it was clear at that time that the overall intensity of infections was decreasing with the expansion of PZQ use through MDAs, and available mapping and diagnostic tools were becoming less useful. Therefore, SCORE also focused on evaluating newly available mapping tools and enhancing and expanding understanding and use of new, more sensitive diagnostic assays. It is hoped that the key findings from these studies and the key messages based on those findings provide a foundation that will contribute to continued operational research to solidify the evidence-base essential for the creation of sound targets and goals, the development of useful guidelines to achieve those goals, and the implementation of successful intervention efforts. The eventual freedom from schistosomiasis in all endemic countries will require coordination across multiple sectors and the combined use of multiple interventions, including infrastructural and economic development. However, continued progress down the winding road to that ultimate goal will require vigorous, unrelenting effort, which we hope will follow on, based on the data and tools generated by SCORE consortium members.
